# *Mycobacterium appelbergii* sp. nov., a Novel Species Isolated from a Drinking Water Fountain in a Rural Community

**DOI:** 10.3390/microorganisms13061259

**Published:** 2025-05-29

**Authors:** Inês Cravo Roxo, Susana Alarico, Ana Fonseca, Daniela Machado, Ana Maranha, Igor Tiago, Raquel Duarte, Nuno Empadinhas

**Affiliations:** 1 CNC—Center for Neuroscience and Cell Biology, University of Coimbra, 3004-504 Coimbra, Portugal; iroxo@cnc.uc.pt (I.C.R.); salarico@cnc.uc.pt (S.A.); anaritafonseca@ua.pt (A.F.); ana.maranha@cnc.uc.pt (A.M.); 2 CIBB—Centre for Innovative Biomedicine and Biotechnology, University of Coimbra, 3004-504 Coimbra, Portugal; 3 PDBEB—PhD Program in Experimental Biology and Biomedicine, Institute for Interdisciplinary Research, University of Coimbra, 3030-789 Coimbra, Portugal; 4 CHVNGE—Centro Hospitalar de Vila Nova de Gaia/Espinho, 4434-502 Vila Nova de Gaia, Portugal; daniela.cunha.machado@ulsge.min-saude.pt; 5 Department of Life Sciences, University of Coimbra, 3000-456 Coimbra, Portugal; itiago@ci.uc.pt; 6 EPIUnit ITR, Instituto de Saúde Pública, University of Porto, 4050-600 Porto, Portugal; raquelafduarte@gmail.com; 7 ICBAS—Instituto de Ciências Biomédicas Abel Salazar, University of Porto, 4050-313 Porto, Portugal; 8 INSA—Instituto Nacional de Saúde Doutor Ricardo Jorge, 4000-404 Porto, Portugal

**Keywords:** nontuberculous mycobacteria, spring water, fountain, biodiversity, genomics, phylogeny, chemotaxonomy

## Abstract

Three isolates of a novel, rapidly growing, non-pigmented *Mycobacterium* species were recovered from the water and runoff of a public fountain in a rural village in central Portugal, formerly used by the local population as a source of drinking water and not accessible to animals. High-quality draft genome sequencing, in silico DNA–DNA hybridization, and phylogenetic analyses confirmed that isolates 21AC1^T^, 21AC14, and 21AC21 represent a previously undescribed species within the genus *Mycobacterium*, forming a distinct phylogenetic lineage closely related to *Mycobacterium wolinskyi*, *Mycobacterium goodii* and *Mycobacterium smegmatis*. MALDI-TOF MS analysis of the type strain 21AC1^T^ revealed a unique spectral profile. A comprehensive polyphasic characterization was performed, including chemotaxonomic analyses of fatty acid and mycolic acid composition, as well as an extensive biochemical characterization. Their susceptibility to 12 antimicrobials was also assessed. The identification and characterization of novel nontuberculous mycobacteria species are of increasing environmental and clinical relevance, as infections by these opportunistic pathogens are on the rise globally. Based on our findings, we propose that isolates 21AC1^T^, 21AC14, and 21AC21 represent a novel species, for which we propose the name *Mycobacterium appelbergii* sp. nov., with the type strain designated as 21AC1^T^ (=BCCM/ITM 501212 = DSM 113570) and the additional two strains as 21AC14 (=BCCM/ITM 501447 = DSM 118402) and 21AC21 (=BCCM/ITM 501448 = DSM 118403).

## 1. Introduction

There are currently around 200 recognized *Mycobacterium* species (https://lpsn.dsmz.de/genus/mycobacterium, accessed on 17 March 2025) most of which are environmental organisms collectively known as nontuberculous mycobacteria (NTM). A subset of these species are opportunistic pathogens capable of causing chronic and severe infections in humans [1,2,3]. NTM are widespread in nature, particularly in water sources, soils, and aerosols. Certain environmental activities and occupational settings can increase the risk of contact with these organisms [4]. Municipal water supplies and plumbing systems represent major reservoirs for human infection, as NTM form biofilms that are highly resistant to standard decontamination procedures, a particular concern for vulnerable populations such as immunocompromised individuals, the elderly, and patients with chronic illnesses [5,6].

Pulmonary infections are the most common clinical manifestation of NTM disease. However, infections may also affect lymph nodes, skin, or soft tissues, as well as becoming disseminated in some cases [7,8,9]. Despite being recognized for over 70 years, NTM continue to pose a growing global health concern [10,11]. Advances in isolation, culturing, and molecular identification techniques have led to a rapid increase in the number of described *Mycobacterium* species. This expanding diversity creates new challenges for clinical management, particularly in diagnosis and in selecting appropriate antimicrobial therapies. Treatment is further complicated due to species-specific differences in antimicrobial susceptibility and intrinsic resistance patterns [12,13,14,15]. These challenges highlight the importance of accurate species identification, robust taxonomic classification, and comprehensive phenotypic and genotypic characterization of these emerging pathogens [16,17].

As part of an ongoing survey of NTM in water and biofilms from sources used by NTM-infected patients, samples were collected from a public water fountain and its runoff. Situated near the home of an individual infected with an *M. avium* strain, this public fountain was historically used by local residents as a source of drinking water and has not been accessible to animals. Analysis of these samples yielded three morphologically identical colonies, designated 21AC1^T^, 21AC14, and 21AC21. Detailed phenotypic and genotypic characterization confirmed that these isolates represent a novel species within the genus *Mycobacterium*.

## 2. Materials and Methods

### 2.1. Water Sampling and Selective Isolation of Mycobacteria

Water samples were collected from a fountain (40.644510, −7.770130) using sterile containers. At the time of collection, the water temperature was 16 °C, and the pH was 6.5. One liter was processed for the selective isolation of NTM, while an additional liter was used for chemical parameter analysis, which was performed by the laboratory CESAB, Mealhada, Portugal (https://www.cesab.pt/, accessed on 20 May 2024). Samples were kept on ice packs and transported to the microbiology and chemistry laboratories within 6 h to ensure sample integrity.

To prevent the overgrowth of contaminants and optimize the recovery of NTM from water samples, decontamination was performed using 0.005% cetylpyridinium chloride (CPC), followed by incubation at room temperature for 20 min. Water samples (200 mL, processed in duplicate) were then filtered through 0.22 μm pore size membranes [18,19]. The membranes were placed onto selective Middlebrook 7H10 agar plates: one supplemented with 1 mg/L malachite green and the other with a cocktail of antimicrobials (PANTA) [20]. The plates were incubated at 30 °C for up to 30 days, with daily monitoring for colony formation. Presumptive NTM colonies were subcultured on Middlebrook 7H10 agar for purification and further characterization.

### 2.2. 16S rRNA Gene Sequencing

Genomic DNA of the three isolates was extracted and purified using the Microbial gDNA Isolation kit (NZYTech, Lisboa, Portugal), which includes an optimized mycobacterial cell lysis step [21]. DNA concentration and quality were assessed using a NanoDrop 2000 spectrophotometer (Thermo Scientific, Waltham, MA, USA). The 16S rRNA gene was amplified by PCR using universal primers 27F (5′-GAGTTTGATCCTGGCTCAG-3′) and 1525R (5′–AGAAAGGAGGTGATCCAGCC-3′) with amplification performed using Supreme NZYTaq DNA polymerase (NZYTech, Portugal). The 16S rRNA gene and whole-genome sequences were obtained through sequencing at Eurofins Genomics (Ebersberg, Germany). The 16S rRNA gene sequence was compared with available sequences in the NCBI database using the BLAST+2.16.0 tool (http://blast.ncbi.nlm.nih.gov/, accessed on 9 September 2024).

### 2.3. Genomic Analysis and Genome Annotation

To determine the phylogenetic position of strains 21AC1^T^, 21AC14, and 21AC21 within the genus *Mycobacterium*, whole-genome sequencing was performed. Genomic libraries were prepared using the Nextera XT Library Preparation kit (Illumina Inc., San Diego, CA, USA), and sequencing was carried out on an Illumina MiSeq platform, generating 2 × 150 nt paired-end reads. For downstream analyses, MetaWRAP v1.3 [22] was used. Quality trimming was performed with the sliding-window operation in TrimGalore v0.5.0 (http://www.bioinformatics.babraham.ac.uk/projects/trim_galore/, accessed on 9 January 2025) using default parameters. Genome assembly was carried out using SPAdes v3.5.0 [23] with default settings and k-mers of 33, 55, and 77 nt. The assembled genome was binned using MetaBat v2.12.1 [24] with default parameters, and a quality assessment was performed using CheckM v1.0.12 [25] under default settings.

### 2.4. Phenotypic Analysis

Monitoring mycobacterial growth in liquid media can be challenging due to cell aggregation, which complicates turbidity measurements. This was mitigated by adding Tween 80 or glycerol to the medium [26]. The ability of isolates 21AC1^T^, 21AC14, and 21AC21 to grow on different solid media, including Middlebrook 7H10 (supplemented with 0.5% glycerol), GPHF agar (DSM 553) (supplemented with 0.5% glycerol), MacConkey agar without crystal violet, Columbia Agar with 5% sheep blood, and Löwenstein–Jensen slants, was assessed. Additionally, for the type strain 21AC1^T^, growth at different temperatures (20, 25, 30, 35, 40 °C) and in the presence of 2 or 5% NaCl was evaluated in Middlebrook 7H9 broth (supplemented with 0.5% glycerol and 0.2% Tween 80).

Strain 21AC1^T^ was tested for catalase and arylsulfatase activities, tellurite reduction, and Tween 80 hydrolysis with assays performed as qualitative tests (presence/absence) following the established protocols cited in references [27,28]. These biochemical assays are key for the differentiation of mycobacterial species, providing qualitative evidence for the presence or absence of specific enzymatic activities. Additional biochemical characteristics of all three isolates were assessed using API Coryne and API 20NE strips (BioMérieux, Marcy-l’Étoile, France) following the manufacturer’s instructions, with incubation at 35 °C for 48 h.

### 2.5. Mycolic and Fatty Acid Analyses (MIDI/GC-MS) and MALDI-TOF MS

The type strain 21AC1^T^ was cultivated on three Middlebrook 7H10 agar plates at 35 °C for 48 h to obtain sufficient biomass for fatty acid and mycolic acid analysis, as well as MALDI-TOF mass spectrometry. These analyses were conducted at the Identification Service of the Leibniz-Institut DSMZ—Deutsche Sammlung von Mikroorganismen und Zellkulturen GmbH, Braunschweig, Germany, following the methodologies outlined at www.dsmz.de/services/microorganisms/biochemical-analysis/cellular-fatty-acids, accessed on 9 October 2024.

### 2.6. Antibiotic Susceptibility Testing

Susceptibility testing was performed according to the Clinical and Laboratory Standards Institute (CLSI) guidelines for rapidly growing mycobacteria [29,30], which applies to the three isolates studied. The antimicrobial agents tested included amikacin (Alfa Aesar, Haverhill, MA, USA), cefoxitin, ciprofloxacin, clarithromycin, doxycycline, imipenem, meropenem, minocycline, tobramycin and trimethoprim/sulfamethoxazole (all from Sigma-Aldrich, St. Louis, MO, EUA), as well as linezolid (Acros Organics, Geel, Belgium), moxifloxacin (TCI Chemicals, Tokyo, Japan). A culture suspension of each strain was prepared by harvesting colonies from Middlebrook 7H10 agar and resuspending them in 5 mL of saline solution to achieve a 0.5 McFarland turbidity standard [20]. The suspensions were vortexed vigorously for 20 s and subsequently diluted 1000-fold before testing, which was conducted within 30 min. Susceptibility testing was performed in sterile 96-well microplates prefilled with Mueller Hinton (MH) medium supplemented with 0.5% OADC. Serial twofold dilution of each antimicrobial agent (from 128 to 0.125 μg/mL) were prepared in the wells. The plates were then inoculated with the diluted bacterial suspension. Minimal inhibitory concentrations (MICs) were defined as the lowest antimicrobial concentration that completely inhibited visible growth, indicated by the absence of a bacterial pellet at the bottom of the well. MICs were determined after 5 days of incubation at 30 °C, except for clarithromycin, which was assessed after 7 days [29,31]. Appropriate controls were included to ensure normal bacterial growth, and all assays were performed in duplicate on two separate days to verify the reproducibility of results.

## 3. Results and Discussion

### 3.1. Sequence Identity and Phylogenetic Analysis

Initial analysis of the 1381 bp 16S rRNA gene sequence from strain 21AC1^T^ showed 98.33% identity with *M. neglectum* CECT 8778, 98.19% with *M. tusciae* CIP 106367 and *M. rufum* JCM 16372, and 98.12% with *M. gilvum* SM 35. As previously reported, 16S rRNA gene sequencing alone often fails to achieve species-level discrimination within the *Mycobacterium* genus [12,13], a limitation also observed in this study.

The draft genome of strain 21AC1^T^ consisted of 7,617,360 bp, with a calculated DNA G+C content of 65.91% and an estimated completeness of 99.93%. The genomes of strains 21AC14 and 21AC21 were assembled to 7,658,160 bp and 7,661,220 bp, respectively (Table S1). The estimated completeness of these genomes was 99.93% and 99.94%, respectively.

**Figure 1:**
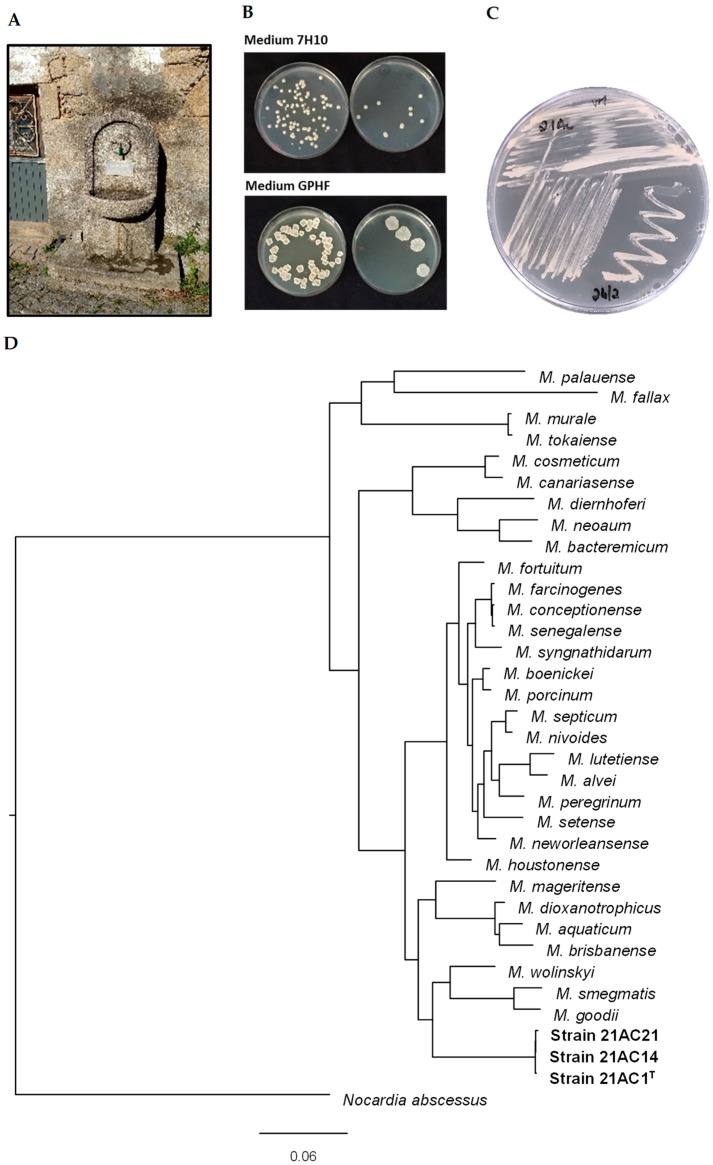
(**A**) Source water fountain located in a rural village in the central region of Portugal. (**B**) Colonies of strain 21AC1^T^ appearing on Middlebrook 7H10 and GPHF (DSM 553) plates after 3 days of incubation at 30 °C. (**C**) Axenic culture of strain 21AC1^T^. (**D**) Phylogenomic tree based on 107 essential single-copy genes inferred from amino acid sequences. Strains 21AC1^T^, 21AC14, and 21AC21, representing the novel *Mycobacterium* species, are shown in bold. *Nocardia abscessus* was used as an outgroup. Accession numbers of reference genomes are provided for each taxon (Table S1). The scale bar represents 0.06 expected changes per site.

Phylogenetic analyses were conducted using the translated coding sequences (CDSs) of the type strains of *Mycobacterium* (retrieved from the NCBI database) (Table S2) and strains 21AC1^T^, 21AC14, and 21AC21. The analysis was based on the comparison of amino acid sequences from 107 single-copy core genes using bcgTree v1.1.0 [32]. Average nucleotide identity (ANI) calculations were performed against phylogenetically related genomes using the EzBioCloud ANI calculator [33]. Phylogenetic analyses confirmed that strains 21AC1^T^, 21AC14, and 21AC21 belong to the same species and are closely related to *M. wolinskyi*, *M. goodii*, and *M. smegmatis* (Figure 1). The tree topology and branch lengths indicate that strain 21AC1^T^ represents a novel species within the genus *Mycobacterium*. This conclusion is further supported by ANIb and digital DNA–DNA hybridization (dDDH) results comparing the genome of strain 21AC1^T^ with those of its closest species relatives (Table 1), and with strains 21AC14 and 21AC21 isolated from the same site (Table S1). The ANI threshold for species delineation (95–96%) [34,35] supports the classification of strain 21AC1^T^ as a distinct species, as it shares ANIb values of approximately 81% with its closest phylogenetic relatives (Table 1). Furthermore, dDDH values between strain 21AC1^T^ and the type strains of *M. wolinskyi*, *M. smegmatis*, and *M. goodii* were 23.6%, 22.1%, and 21.8%, respectively (Table 1), which are well below the 70% threshold for species discrimination [34]. The dDDH value among strains 21AC1^T^, 21AC14, and 21AC21 was 100% (Table S1), confirming their classification as members of the same species. Additionally, analysis using the Type (Strain) Genome Server (TYGS) further corroborated that the three isolates belong to a novel species within the genus *Mycobacterium*.

### 3.2. Nucleotide and Genome Sequence Accession Numbers

The 16S rRNA gene sequences of *Mycobacterium appelbergii* strains 21AC1^T^, 21AC14, and 21AC21 have been deposited into GenBank under the accession numbers OP795714, PV139203, and PV130038, respectively. The assembled genomes of these three strains were annotated using the NCBI Prokaryotic Genomes Annotation Pipeline (PGAP). The whole-genome shotgun (WGS) projects have been deposited at DDBJ/ENA/GenBank under accession numbers JAMQTH000000000, JBLKDU000000000, and JBLKDT000000000, respectively.

### 3.3. Chemical Analysis of the Water Source of Strain Isolation

The chemical composition and concentrations of selected metals and mineral salts in the water source are summarized in Table 2, in accordance with established water quality parameters. Previous studies have linked certain constituents, particularly molybdenum and calcium levels [36], trace molybdenum and vanadium salts [37], and the combined presence of molybdenum, vanadium, and sulphate, to an increased incidence of NTM pulmonary infections, especially in cystic fibrosis patients [38]. In the present work, the concentrations of molybdenum and vanadium fell well within the maximum limits set by both the German Environmental Protection Agency and the US Environmental Protection Agency for drinking water [39,40].

### 3.4. Physiological and Chemotaxonomic Analysis

Strain 21AC1^T^ grew in Middlebrook 7H9 broth supplemented with 0.5% of glycerol across a temperature range of 20–35 °C, with optimal growth observed between 30 and 35 °C. A comparable optimal temperature range was identified for all three isolates cultured on solid Middlebrook 7H10 medium (Table 3). At 25–35 °C, the strains formed non-pigmented, light beige colonies within 2–3 days. Colony morphology varied with the growth medium: on Middlebrook 7H10, colonies appeared small and round, whereas on GPHF agar they appeared larger, rough, and dry (Figure 1). Such phenotypic variation in colony morphology is common and can be influenced by environmental factors such as medium composition, temperature, and host interactions. This variability is well-documented in species like *M. avium* and *M. abscessus*, which can switch between morphotypes with distinct characteristics [41]. These morphological shifts can be clinically relevant, as they can affect host–pathogen interactions and antibiotic susceptibility [42].

Strain 21AC1^T^ tested positive for catalase and arylsulfatase activities, tellurite reduction, and Tween 80 hydrolysis. Based on API Coryne and API 20E strip results, all three strains showed positive reactions for nitrate reductase, pyrazinamidase, alkaline phosphatase, β-glucosidase, esculin hydrolysis, urease, and acetoin production (Voges–Proskauer test). None of the carbon sources included in the API strips were utilized by the strains under the tested conditions (Table 3). In contrast, *M. fortuitum*, used as a control in this study, utilized only L-arabinose as a sole carbon source, consistent with previous reports [28]. The same study also reported that *M. porcinum* could use inositol as a sole carbon source [28]. Furthermore, other mycobacterial species, such as *M. barrassiae* and *M. moriokaense*, demonstrated the ability to utilize several sole carbon sources when tested using API Coryne and API 20E strips [43].

Fatty acid analysis was conducted using the MIDI system and GS/MS (Table 4). Strain 21AC1^T^ displayed a fatty acid methyl ester (FAME) profile predominantly composed of the saturated fatty acid C16:0, followed by the unsaturated FAMEs C16:1ω6c and C18:1ω9c, along with the characteristic tuberculostearic acid (TBSA) 10Me-18:0. This fatty acid composition closely resembles the profiles reported for *M. smegmatis*, *M. fortuitum*, and *M. genavense* [44,45,46].

Mycolic acids were identified by mass spectrometry, and their relative abundances were calculated from the total pool of detected mycolates (Table 4). In strain 21AC1^T^, the predominant species was the α-mycolate C_77_H_150_O_3_ (36.25% relative abundance), followed by C_75_H1_46_O_3_ (10.78%) and the oxygenated mycolate C_79_H_154_O_4_ (9.92%). Based on exact masses, 81.5% of the mycolates were classified as α-mycolates and 18.5% as oxygenated (inferred to be epoxy-mycolates) [47]. Comparable profiles have been reported for *M. goodii*, *M. wolinskyi*, and *M. smegmatis* by HPLC analysis [48,49]. In particular, *M. smegmatis* produces three main mycolate classes: (i) α-mycolic acids (50–60% of total); (ii) α′-mycolates, a shorter variant found in some rapidly growing mycobacteria (RGM); and (iii) epoxy-mycolates, containing an epoxide ring and largely confined to the *M. fortuitum-M. smegmatis* group [47]. Both α′- and epoxy-mycolates each account for roughly 15–30% of the mycolate pool [50,51]. Chain lengths for α′-mycolates are typically C_62_–C_64_ [52,53,54], whereas α- and epoxy-mycolates range from C_77_ to C_80_ [55]. Finally, MALDI-TOF Biotyper analysis of strain 21AC1^T^ yielded a score of 1.2, corresponding to “no reliable classification”.

**Table 3:** Growth characteristics and biochemical features of *Mycobacterium appelbergii* strains 21AC1^T^, 21AC14, and 21AC21, compared with closely related species.

		21AC1^T^	21AC14	21AC21	*M. wolinskyi* [31,48]	*M. goodii* [31]	*M. smegmatis* [31,48]
Optimal growth on solid media	7H10	30–35 °C	30–37 °C	30–37 °C	30–45 °C	30–45 °C	30–45 °C
	GPHF	25–30 °C	ND	ND	NA	NA	NA
Growth on Middlebrook 7H9	20 °C	+	ND	ND	NA	NA	NA
	25 °C	+	ND	ND	NA	NA	NA
	30 °C	+	ND	ND	+	+	NA
	35 °C	+	ND	ND	+	+	NA
	42 °C	-	ND	ND	+	+	NA
5% NaCl tolerance	30 °C	+	ND	ND	+	+	+
Catalase	RT	+	ND	ND	NA	NA	+
	t = 0, 68 °C	+	ND	ND	+	+	NA
	t = 20, 68 °C	+	ND	ND	+	+	NA
Growth in McConkey agar (without crystal violet)		-	ND	ND	+	+	+
Tween 80 hydrolysis		+	ND	ND	NA	NA	NA
Arylsulfatase		+	ND	ND	-	-	-
Iron uptake		-	ND	ND	+	+	+
Tellurite reduction		+	ND	ND	NA	NA	NA
Nitrate reductase		+	+	+	+	+	+
Pyrazinamidase		+	+	+	NA	NA	NA
Pyrrolidonylarylamidase		-	-	-	NA	NA	NA
Alkaline phosphatase		+	+	+	NA	NA	NA
β-Glucuronidase		-	-	-	NA	NA	NA
β-Galactosidase		-	-	-	NA	NA	NA
β-Glucosidase		+	+	+	NA	NA	NA
*N*-Acetyl-β-glucosaminidase		-	-	-	NA	NA	NA
Esculin		+	+	+	NA	NA	NA
Urease		+	+	+	NA	NA	NA
Gelatinase		-	-	-	NA	NA	-
Arginine dihydrolase		-	-	-	NA	NA	NA
Lysine decarboxylase		-	-	-	NA	NA	NA
Ornithine decarboxylase		-	-	-	NA	NA	NA
Citrate		-	-	-	+	-	+-
H_2_S production		-	-	-	NA	NA	NA
Tryptophan deaminase		-	-	-	NA	NA	NA
Indole production		-	-	-	NA	NA	NA
Acetoin production		+	+	+	NA	NA	NA
Utilization of carbon sources							
D-Glucose		-	-	-	NA	NA	+
D-Mannitol		-	-	-	+	+	+
Inositol		-	-	-	+	+	+
D-Sorbitol		-	-	-	+	+	+
L-Rhamnose		-	-	-	+	+	+
D-Saccharose		-	-	-	NA	NA	NA
Amygdaline		-	-	-	NA	NA	NA
L-Arabinose		-	-	-	+	+	+
Ribose		-	-	-	NA	NA	NA
Xylose		-	-	-	+	+	+
Maltose		-	-	-	NA	NA	NA
Lactose		-	-	-	NA	NA	NA
Glycogen		-	-	-	NA	NA	NA

+ (positive result for the determined parameter); - (negative result for the determined parameter); ND (Not Determined): assay not performed; NA (Not Available): data unavailable in the referenced literature.

**Table 4:** Relative abundance (%) of cellular fatty acids and mycolic acids identified in strain 21AC1^T^. Fatty acids present at <1.0% were omitted.

Fatty Acid	(%)	Mycolic Acid	(%)
Saturated FAME		C_62_H_122_O_3_	7.17
C14:0	5.2	C_64_H_126_0_3_	3.2
C16:0	34.1	C_75_H_146_0_3_	10.78
C18:0	2.4	C_76_H_148_0_3_	9.02
Unsaturated FAME		C_77_H_150_0_3_	36.25
C16:1 ω6c	16.3	C_78_H_152_0_3_	7.12
C18:1 ω9c	27.7	C_77_H_150_0_4_	8.57
Tuberculostearic acid [TBSA] FAME		C_79_H_154_0_3_	7.97
10Me-18:0	14.3	C_79_H_154_0_4_	9.92

FAME, fatty acid methyl ester.

### 3.5. Antibiotic Susceptibility Profiles

Although the pathogenic potential of strain 21AC1^T^ remains unknown, infections by the closely relate species *M. wolinskyi*, *M. goodii*, and *M. smegmatis*, historically rare in humans, are increasingly reported, particularly in nosocomial settings [56,57,58,59,60,61]. Our study followed the CLSI-recommended panels for NTM and rapidly growing mycobacteria (RGM) susceptibility testing. The antimicrobial susceptibility profiles of the three isolates belonging to the novel species are summarized in Table 5.

Strain 21AC1^T^ was susceptible to 10 of the 12 tested antimicrobials and exhibited intermediate susceptibility to imipenem and doxycycline. Strains 21AC14 and 21AC21 exhibited intermediate susceptibility to ciprofloxacin and tobramycin, whereas strain 21AC21 was resistant to imipenem (Table 5). Intermediate susceptibility to doxycycline has previously been reported for *M. wolinskyi*, *M. goodii*, and clinical isolates of *M. smegmatis* [48]. Neither strain 21AC1^T^ nor 21AC14 showed resistance to tobramycin, in contrast to *M. wolinskyi*, which has been reported to exhibit resistance to this antimicrobial [48].

## 4. Conclusions

The present study describes the isolation and characterization of three isolates of a novel, rapidly growing non-pigmented NTM, for which the name *Mycobacterium appelbergii* sp. nov. is proposed. All three isolates, recovered from the water of a public fountain, shared a coherent suite of phenotypic traits and genomic markers that clearly differentiate them from three nearest relatives, *M. goodii*, *M. wolinskyi*, and *M. smegmatis.* Comprehensive phylogenomic, biochemical, and chemotaxonomic analyses uniformly support their status as a distinct species.

By expanding the known diversity of environmental mycobacteria, the identification of *M. appelbergii* underscores the role of water supply systems as reservoirs for NTM. Although this work focused on taxonomic characterization, the isolation of *M. appelbergii* from a drinking water source highlights the need for follow-up studies on its ecological persistence and potential clinical relevance. This foundational data lays the groundwork for future investigations of this newly recognized species.

This work provides critical insight into the hidden diversity of environmental NTM and underscores the importance of monitoring public water sources as potential reservoirs of opportunistic pathogens.

## 5. Description of *Mycobacterium appelbergii* sp. nov.

*Mycobacterium appelbergii* (*ap.pel.berg’i.i.* N.L. adj. *appelbergii*, in honor of Rui Appelberg, in recognition of his significant scientific contributions to the understanding of the immune response, vaccine development, and immune pathology associated with *Mycobacterium avium* and *Mycobacterium tuberculosis* infections). *Mycobacterium appelbergii* is a non-motile, non-spore-forming bacillus. Colonies are non-pigmented (light beige), appearing within 2–3 days at temperatures 25–35 °C, with distinct morphologies on different media: small and round on Middlebrook 7H10 agar, and larger, irregular, and dry on GPHF agar. The bacteria grow on Middlebrook 7H9 broth within approximately 24 h at temperatures ranging from 20 °C to 35 °C, with an optimal growth temperature at about 30 °C. No growth occurs at 40 °C. The species tolerates up to 5% NaCl. *Mycobacterium appelbergii* is biochemically positive for catalase (room temperature and 68 °C), arylsulfatase, tellurite reduction, Tween 80 hydrolysis, nitrate reductase, pyrazinamidase, alkaline phosphatase, β-glucosidase, esculin hydrolysis, urease, and acetoin production. The major cellular fatty acids include C16:0, C16:1ω6c, C18:1ω9c, and 10Me-18:0 (tuberculostearic acid, TBSA). The predominant mycolic acid is C_77_H_150_0_3_. Antimicrobial susceptibility testing indicates that *M. appelbergii* is susceptible to amikacin, cefoxitin, clarithromycin, linezolid, meropenem, minocycline, moxifloxacin, and trimethoprim-sulfamethoxazole (TMP-SMX), exhibits intermediate susceptibility to doxycycline, and exhibits variable susceptibility to ciprofloxacin (S-I), tobramycin (S-I), and imipenem (I-R). The genomic DNA G+C content ranges from 65.88–65.91 mol%. Three strains were isolated from water samples from a public fountain and its runoff. The type strain, 21AC1^T^, has been deposited in the Deutsche Sammlung von Mikroorganismen und Zellkulturen (DSMZ), Braunschweig, Germany, as DSM 113570, and in the Belgium Coordinated Collections of Microorganisms (BCCM) Mycobacteriology Unit, Institute of Tropical Medicine, as BCCM/ITM 501212.

## Figures and Tables

**Table 1:** Genomic-based comparison between strain 21AC1^T^ and type strains of closely related species *Mycobacterium wolinskyi*, *Mycobacterium smegmatis*, and *Mycobacterium goodii*.

Genome A	21AC1^T^ vs.			*M. goodii * vs.		*M. wolinskyi * vs.
Genome B	*M.* *wolinskyi*	*M. smegmatis*	*M.* *goodii*	*M.* *wolinskyi*	*M.* *smegmatis*	*M.* *smegmatis*
dDDH values in % *	23.6 (30.4)	22.1 (27)	21.8 (26.6)	23.8 (33.4)	34.9 (65.9)	23.7 (32.6)
OrthoANIu value (%)	80.76	79.34	79.11	81.00	88.16	81.07
Average aligned length (bp)	3,170,245	2,901,392	2,802,236	3,105,041	3,862,094	2,991,255
Genome A coverage (%)	41.62	38.09	36.79	46.06	57.29	39.88
Genome B coverage (%)	42.26	41.48	41.57	41.39	55.22	42.77
Type strains	21AC1^T^	*M. wolinskyi* ATCC 700010		*M. goodii* ATCC 700504	*M. smegmatis* NCTC 8159	
Genome length (bp)	7,617,360	7,501,080		6,741,281	6,994,140	

* The first value corresponds to dDDH formula d4 (also known as GGDC formula 2), which represents the sum of all identities found in high-scoring segment pairs (HSPs) divided by the overall HSP length. The value in parentheses corresponds to dDDH formula d0 (also known as GGDC formula 1), which represents the length of all HSPs divided by the total genome length. dDDH formula d4 is independent of genome size and is more robust when analyzing incomplete genomes.

**Table 2:** Physicochemical parameters of the water from the public fountain.

Parameters/Method *	Result	Unit	U (%)	MAC
pH	6.5 at 20 °C	Sorensen Scale	±0.2; ±0.1	∙∙∙
Alkalinity	33	mg CaCO_3_ L^−1^	±11; ±1.6	∙∙∙
Calcium	11	mg Ca L^−1^	±16; ±2.9	∙∙∙
Conductivity	136	µS/cm, at 20 °C	±4; ±1.1	∙∙∙
Total hardness	33	mg CaCO_3_ L^−1^	∙∙∙	∙∙∙
Molybdenum	<10	μg Mo L^−1^	±20; ±7.5	50–70
Vanadium	<0.010	mg V L^−1^	∙∙∙	∙∙∙
Nitrate	19	mg NO_3_ L^−1^	±27; ±(m)	50
Nitrite	<0.010	mg NO_2_ L^−1^	±25; ±(m)	0.5
Chloride	8.6	mg Cl^−^ L^−1^	±26; ±(m)	250

* Selected parameters from the water analysis conducted by CESAB (Centro de Serviços do Ambiente, https://www.cesab.pt/), a water quality control service in Portugal. **U**, expanded global measurement uncertainty value. MAC, Maximum Allowable Concentration in drinking water (based on EU, WHO, or EPA guidelines).

**Table 5:** Antimicrobial susceptibility of *Mycobacterium appelbergii* strains.

Antimicrobial	MIC (µg/mL)		
	Strain 21AC1^T^	Strain 21AC14	Strain 21AC21
Amikacin	0.25 ^S^	0.5 ^S^	0.25 ^S^
Cefoxitin	0.25 ^S^	2 ^S^	2 ^S^
Ciprofloxacin	0.5 ^S^	2 ^I^	2 ^I^
Clarithromycin	<0.125 ^S^	<0.125 ^S^	<0.125 ^S^
Imipenem	16 ^I^	16 ^I^	32 ^R^
Linezolid	1 ^S^	0.25 ^S^	0.25 ^S^
Meropenem	0.25 ^S^	0.125 ^S^	0.25 ^S^
Minocycline	0.25 ^S^	0.5 ^S^	0.5 ^S^
Moxifloxacin	0.25 ^S^	0.5 ^S^	0.25 ^S^
Tobramycin	1 ^S^	4 ^I^	4 ^I^
Doxycycline	2 ^I^	2 ^I^	2 ^I^
TMP-SMX	0.5–9.5 ^S^	<0.125–2.375 ^S^	<0.125–2.375 ^S^

MIC—minimum inhibitory concentration; ^S^—Susceptible; ^I^—Intermediate, ^R^—Resistant. Grey shadow tones represent variable susceptibility among the three isolates. Breakpoint interpretation was performed according to the [30].

## Data Availability

The original contributions presented in this study are included in the article/Supplementary Material. Further inquiries can be directed to the corresponding author.

## Supplementary Materials

The following supporting information can be downloaded at: https://www.mdpi.com/article/10.3390/microorganisms13061259/s1, Table S1: Genomic comparison of type strain 21AC1^T^ and strains 21AC14 and 21AC21. Table S2. Accession numbers of reference genomes used in the phylogenetic analysis.

## Abbreviations

NTM, nontuberculous mycobacteria; OADC, oleate-albumin-dextrose-catalase; LJ, Löwenstein–Jensen; MIC, Minimal inhibitory concentration; MALDI-TOF, Matrix-assisted laser desorption/ionization time-of-flight mass spectrometry; DDH, DNA–DNA hybridization.
