# Supplementary material for: Mycobacterium appelbergii sp. nov., a Novel Species Isolated from a Drinking Water Fountain in a Rural Community
Source: Microorganisms. 2025 May 29;13(6):1259. doi: 10.3390/microorganisms13061259 (PMC12195061; doi:10.3390/microorganisms13061259)
Supplement: Supplementary file 1 [file microorganisms-13-01259-s001.zip › microorganisms-3648450-supplementary.pdf]

Supplementary Data

Table S1: Genomic comparison of type strain 21AC1^T and strains 21AC14 and 21AC21.

**Genome A**	**21AC1^T**	**21AC1^T**	**21AC14**
**Genome B**	**21AC21**	**21AC14**	**21AC21**
dDDH values in % *	100	100	100
OrthoANIu value (%)	99.98	99.99	99.99
Average aligned length (bp)	6,012,913	6,331,088	6,201,778
Genome A coverage (%)	78.49	82.67	80.98
Genome B coverage (%)	78.94	83.11	80.95
Strains	**21AC1^T**	**21AC14**	**21AC21**
Genome length (bp)	7,617,360	7,658,160	7,661,220

* The value corresponds to dDDH formula d4 (also known as GGDC formula 2), which represents the sum of all identities found in high-scoring segment pairs (HSPs) divided by the overall HSP length. This formula is independent of genome size and is more robust when analysing incomplete genomes.

Table S2. Accession numbers of reference genomes used in the phylogenetic analysis.

Strain	Accession number	Strain	Accession number
M. tokaiense[T]	NZ_UGQT00000000	*M. farcinogenes*[T]	CCAY00000000
M. murale[T]	GCA_010722995	*M. senegalense*[T]	LDCO00000000
M. palauense[T]	GCA_002592005	*M. conceptionense*[T]	NZ_LQOP00000000
M. fallax[T]	NZ_LQOJ00000000	*M. syngnathidarum*[T]	NZ_MLCL00000000
M. diernhoferi[T]	PDCR00000000	*M. porcinum*[T]	MVIG00000000
M. neoaurum[T]	JMDW00000000	*M. boenickei*[T]	PDCO00000000
M. bacteremicum[T]	MVHJ00000000	*M. neworleansense*[T]	CWKH00000000
M. cosmeticum[T]	CCBB00000000	*M. setense*[T]	JTJW00000000
M. canariasense[T]	NZ_LQOL00000000	*M. nivoides*[T]	GCF_003855255
M. mageritense[T]	CCBF00000000	*M. septicum*[T]	NZ_CBMO010000000
M. dioxanotrophicus[T]	GCF_002157835	*M. alvei*[T]	GCA_010727325
M. aquaticum[T]	MVHF00000000	*M. lutetience*[T]	GCF_017876775
M. brisbanense[T]	BCSX00000000	*M. peregrinum*[T]	NZ_LQPP00000000
M. smegmatis[T]	NZ_LN831039	*M. fortuitum*[T]	ALQB00000000
M. goodii[T]	GCF_022370755	*M. houstonense*[T]	FJVO00000000
M. wolinskyi[T]	NZ_LQQA00000000	*Nocardia abscessus*[T]	GCA_000308455
